# Individual-based network model for Rift Valley fever in Kabale District, Uganda

**DOI:** 10.1371/journal.pone.0202721

**Published:** 2019-03-05

**Authors:** Musa Sekamatte, Mahbubul H. Riad, Tesfaalem Tekleghiorghis, Kenneth J. Linthicum, Seth C. Britch, Juergen A. Richt, J. P. Gonzalez, Caterina M. Scoglio

**Affiliations:** 1 Zoonotic Disease Coordination Office (ZDCO), National One Health Platform (NOHP), Ministry of Health, Kampala, Uganda; 2 Department of Electrical and Computer Engineering, College of Engineering, Kansas State University, Manhattan, KS, United States of America; 3 Department of Diagnostic Medicine/Pathobiology, College of Veterinary Medicine, Kansas State University, Manhattan, KS, United States of America; 4 USDA-Agricultural Research Service Center for Medical, Agricultural, and Veterinary Entomology, Gainesville, FL, United States of America; Universidad Rey Juan Carlos, SPAIN

## Abstract

Rift Valley fever (RVF) is a zoonotic disease, that causes significant morbidity and mortality among ungulate livestock and humans in endemic regions. In East Africa, the causative agent of the disease is Rift Valley fever virus (RVFV) which is primarily transmitted by multiple mosquito species in *Aedes* and *Mansonia* genera during both epizootic and enzootic periods in a complex transmission cycle largely driven by environmental and climatic factors. However, recent RVFV activity in Uganda demonstrated the capability of the virus to spread into new regions through livestock movements, and underscored the need to develop effective mitigation strategies to reduce transmission and prevent spread among cattle populations. We simulated RVFV transmission among cows in 22 different locations of the Kabale District in Uganda using real world livestock data in a network-based model. This model considered livestock as a spatially explicit factor in different locations subjected to specific vector and environmental factors, and was configured to investigate and quantitatively evaluate the relative impacts of mosquito control, livestock movement, and diversity in cattle populations on the spread of the RVF epizootic. We concluded that cattle movement should be restricted for periods of high mosquito abundance to control epizootic spreading among locations during an RVF outbreak. Importantly, simulation results also showed that cattle populations with heterogeneous genetic diversity as crossbreeds were less susceptible to infection compared to homogenous cattle populations.

## Introduction

Rift Valley fever (RVF) is a zoonotic mosquito-borne disease caused by Rift Valley fever virus (RVFV; *Phlebovirus*: Bunyaviridae). It severely affects ungulate livestock and wildlife but can also affect humans in RVF-endemic regions of sub-Saharan Africa and parts of the Arabian Peninsula [1–2]. Major RVF outbreaks have been reported in Egypt (1977, 2003), Kenya (1997, 1998, 2006, 2007), Tanzania (2007), Somalia (2007), Saudi Arabia and Yemen (2000–2001), Sudan (2007), Senegal (2013–2014), Mauritania (2010, 2012, 2013–2014), Uganda (2016), and Niger (2016) [3–11]. Potential economic impact and public and veterinary health burdens due to RVF outbreaks have been documented [3, 12–15]. Persistent heavy rainfall causing flooding is the most prominent precursor of RVF epizootics in East Africa, due to flooding- ground pools stimulating massive emergence of transovarially RVFV-infected *Aedes* mosquitoes [16–17]. The transmission cycle of RVFV initiates as the virus is introduced into livestock by competent mosquitoes during blood feeding [2, 18–19]. However, the West Africa epizootic regions do not experience transmissions linked to elevated rainfall [20]. In these areas, RVFV is most likely spread via movements of infected livestock from endemic areas. Livestock trading across different market areas may include infected cows that could disperse the virus in the presence of competent mosquitoes [21]. Patterns of recent RVF activity in Uganda support the hypothesis of RVFV spread linked to the cattle trade [22]. This event in Uganda underscored the need to develop effective operational surveillance and mitigation strategies to reduce or prevent spread among cattle operation locations. Mathematical/epidemic models offer the possibility to investigate RVFV and other infectious disease dynamics through time, and may be used to devise mitigation strategies [23].

Using epidemic models, potential impact of an RVFV outbreak can be quantitatively assessed from simulations. The importance of space in RVF endemicity in West Africa was demonstrated by placing a mosquito habitat under surveillance to find the triggering point for an RVF epidemic [24]. Models showed animals can infect humans and mosquitoes, however humans cannot infect mosquitoes or livestock [25]. A Bayesian spatial model for RVF spreading was proposed to investigate environmental drivers that alter host and vector distributions [26]. In Kenya, an ecological niche model was formulated to predict the distribution of RVF vector species under climate change [27].

An individual-level network model was proposed to demonstrate the effect of network topologies based on inter-farm cattle movement in the United States [23]. Two separate kernel functions—exponential and power-law kernels—were used to model cattle movement within and among farms in Riley County, Kansas. Between simulations with two kernel functions, widespread epizootics from the power-law model were revealed, because cows were allowed to move to distant farms. In contrast, the exponential model greatly restricted cattle movement to more proximal farms, reducing spread of the virus.

In this study, we developed a network-based epidemic transmission model to perform simulations. Simulation results provided an opportunity to investigate patterns of RVFV across locations in the Kabale District, Uganda. We built upon a previous individual-based network model to investigate RVFV epidemiology in the Kabale District using 2012 livestock data from UBOS [24]. This model considered livestock as a spatially explicit factor in an individual-based network representing different locations with specific mosquito and environmental factors. Our goal was to investigate changes in the epidemic size (total number of infected cows) for varying mosquito abundance, different initial conditions (single- or multiple-outbreak locations), cattle breed (indigenous or exotic), and cattle movement. We were able to suggest several mitigation strategies to check/reduce RVFV spread using simulation results from the individual-based network model.

## Materials and methods

### Modeling framework

**Fig 1:**
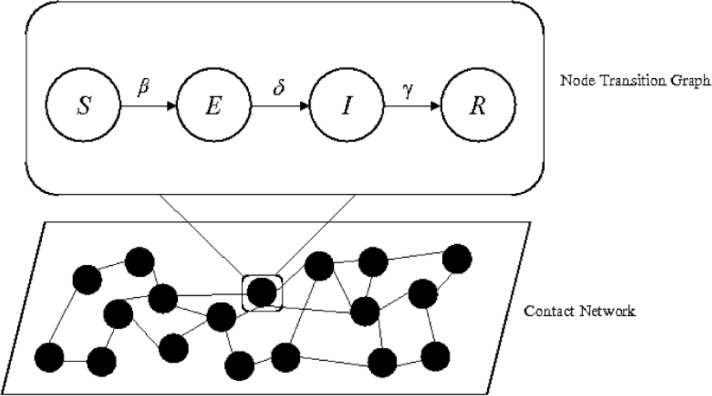
Diagram of an individual-based network model that consists of a node transition graph and a contact network. Circles in the node transition graph represent the four compartments *susceptible (S)*, *exposed (E)*, *infectious (I)*, and *recovered (R)* of a node (i.e., of an individual cow), and arrows between the compartments show the direction of transition for each node (cow) with rates driven by parameters *β* (transmission rate), *δ* (infectious rate), and *γ* (recovery rate). Circles in the contact network, in turn, represent individual cows (i.e., nodes), and black lines linking circles represent opportunities for RVFV transmission.

RVFV modeling framework consists of two parts, a node transition graph and a contact network. The node transition graph consists of four compartments—susceptible (*S*), exposed (*E*), infectious (*I*), and recovered (*R*). Each individual cow can be in only one of these four compartments and rates of transitions between compartments are driven by parameters *β* (transmission rate), *δ* (infectious rate), and *γ* (recovery rate). **Fig 1** represents the conceptual core of the spread model, showing the sequence of the progression of the RVFV infection in a cow (node) through four compartments.

The contact network consists of the total number of cows (*N*) in the network, each represented by a small circle (i.e., node), and a black line linking (i.e., links/edges) two nodes when an opportunity for transmission of RVFV between two cows (via the bite of an infectious mosquito) arises (**Fig 1**). A link between two nodes occurs if (a) virus transfer is possible between them (i.e., when one is infectious and the other susceptible), and (b) if they are in physical proximity; and virus transfer ultimately happens via infected local mosquito species competent for transmission of RVFV. Thus, links connecting nodes represent possibilities of RVFV transmission from an infected cow to a susceptible cow by a mosquito.

In the network model, the infection can spread if a susceptible node (i.e., a susceptible cow) is in physical proximity with at least one infectious node. Specifically, one infectious cow (node 1) will be able to transmit RVFV to a susceptible cow (node 2) only if there are enough RVFV-competent mosquitoes to first bite the infectious cow (node 1) then, after an appropriate period of time for the virus to disperse and replicate in the mosquito, bite a susceptible cow (node 2) [23]. As stated before, links between cows in the network represent the possibility of virus transfer via mosquitoes once cows are in physical proximity for a sufficient period.

We explicitly modeled cows and mosquitoes were included in an aggregated way with a transmission parameter from an infectious animal to a susceptible one. This transmission parameter was directly proportional to vectorial capacity, which included mosquito abundance, survival rate, vector competence, and feeding patterns [23]. Once a susceptible (*S*) node was in physical proximity of an infectious node, virus transfer took place with transmission rate *β*, and moved the cow into the exposed (*E*) compartment. If a susceptible cow had *Y*_*i*_ infectious neighbors, then the probability of the susceptible cow to receive a virus transmission was *βY*_*i*_. Therefore, the total rate at which susceptible cows became infected was proportional to the number of infectious cows in the neighborhood and the vectorial capacity of available mosquito vectors. The transition of the cow from the exposed compartment (*E*) to the infectious (*I*) compartment took place at rate *δ*, and represented the time the pathogen will take, once it entered into the host body, to replicate enough for the cow to become infectious–i.e., capable of infecting a naïve mosquito. Infectious cows finally transferred to the recovered/removed compartment (*R*) with rate *γ*. We did not consider disease-induced mortality; the endpoint in the simulation for an individual cow (node) was reached when it entered the *R* compartment.

Parameters *δ* and *γ* were specified according to the literature. For our simulations, we invariably used the value of *δ = 0*.*33 day*^*-1*^ (3-day incubation period) and *γ = 0*.*14 day*^*-1*^ (7- day recovery period) [23]. Transmission rate *β* is dependent upon vector abundance as well as various environmental factors and, thus, cannot be expressed with a single value. Therefore, we used a range of *β* to explore various magnitudes of environmental factors as well as mosquito abundance. The transmission rate was proportional to the realized vectorial capacity of competent mosquito species likely to be present in the study area.

After developing the individual-based *SEIR* network model for the Kabale District, we carried out extensive simulations using a Generalized Epidemic Modeling Framework (GEMF) developed by the Network Science and Engineering (NetSE) group at Kansas State University [18]. In the SEIR model, based on GEMF, infection processes were Poisson processes independent of each other. The node-level Markov process for node *i*, *i* = 1, 2, …*N*, was expressed as:

$$\mathrm{Pr}[x_{i}(t+\Delta t)=1|x_{i}(t)=0,X(t)]=\beta Y_{i}\Delta t+o(\Delta t),$$

$$\mathrm{Pr}[x_{i}(t+\Delta t)=2|x_{i}(t)=1,X(t)]=\lambda \Delta t+o(\Delta t),$$

$$\mathrm{Pr}[x_{i}(t+\Delta t)=3|x_{i}(t)=2,X(t)]=\delta \Delta t+o(\Delta t),$$

where *x*_*i*_ = 0, 1, 2, or 3, which corresponded to node *i* being in the susceptible, exposed, infectious, or recovered/removed state, respectively [9]. The value *X* (*t*) was the joint state of all nodes–the network state–at time *t*. This model used a GEMF because it was individual-based, which provided more accurate predictions than meta-population models [23].

#### Geographic structure and movement in the cattle contact network (CCN)

We modeled the cattle movement network based on the local trading system for the Kabale District while considering two different networks depending upon the relative susceptibility of exotic and indigenous cattle. The cattle contact network consisted of 20,806 cows (*N*) unevenly distributed across 22 locations in the Kabale District of Uganda in 2012 (**Table 1**), which is approximately 1,679 km^2^ (648 sq mi) in the western region of Uganda (UBOS) [24].

**Table 1:** Cows in different locations in the Kabale District; this data set was derived from the UBOS Statistical Report 2012, Kabale District [24].

Location	Number of Exotic Cows	Number of Indigenous Cows	Total
**Bubale**	1721	1580	3301
**Bufundi**	74	804	878
**Buhara**	215	837	1052
**Bukinda**	61	268	329
**Butanda**	24	403	427
**Hamurwa**	267	1083	1350
**Hamurwa T/C**	116	582	698
**Ikumba**	141	845	986
**Kabale Municipality**	336	600	936
**Kaharo**	87	578	665
**Kamuganguzi**	367	526	893
**Kamwezi**	187	1623	1810
**Kashambya**	68	721	789
**Katuna T/C**	304	271	575
**Kitumba**	187	692	879
**Kyanamira**	361	719	1080
**Maziba**	141	427	568
**Muhanga T/C**	42	276	318
**Muko**	38	872	910
**Rubaya**	180	1008	1188
**Ruhija**	8	382	390
**Rwamucucu**	71	713	784
**Total**	4996	15810	20806

**Fig 2:**
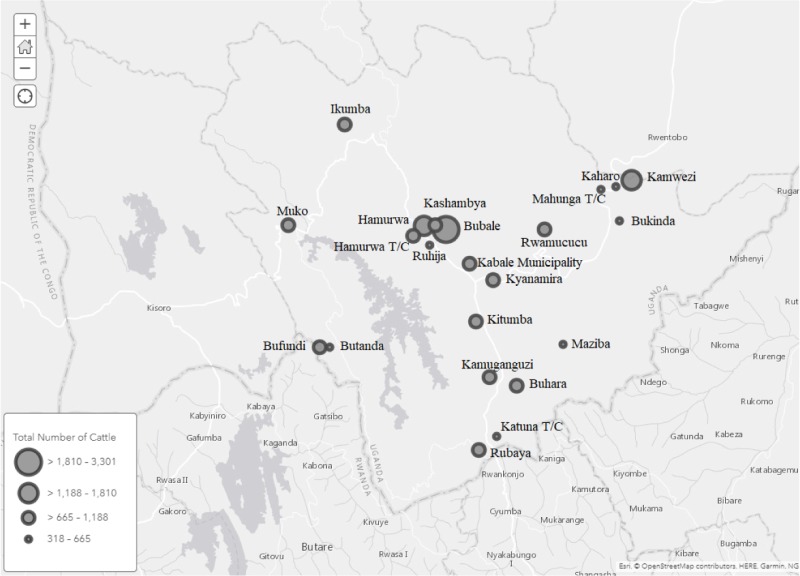
Locations of cattle contact networks in the Kabale District; circles represent center of each location. Circles diameters are scaled to total number of cattle in each location. Bigger size of circle represents greater numbers of cattle within a location.

Data from a sub-county, municipality (Kabale) or town council (Hamurwa, Muhanga, and Katuna) boundary (**Table 1**) represented locations. We extracted the longitude and latitude of the centroid of each location from Google Maps to display in a GIS as shown in **Fig 2**. We have further addressed each of them only by location without any distinction.

To capture actual movement of cows in Uganda, we treated contact among cows (not physical; rather, implicit contact via mosquitoes) differently depending on geographic scale: cows were assumed to move freely within each location, while their movement was restricted between locations. We assumed each cow had equal connection probability to all other individual cows in that location via mosquitoes because of their proximity. We found that an Erdos-Renyi network best represented this relationship among cows within locations, where each cow had equal probability of connectivity (we assumed probability 0.7 for a connected network) to any other cow [28, 29, 30].

Transmission of RVFV from one location to another can happen via movement of cows for economic reasons, most commonly through sales at local market places. Therefore, contact among cows, i.e., the possibility of virus transfer, was weighted in proportion to the distances between locations for the local trading system. We accomplished this weighting with an exponential distance kernel, expressed as *e*^*-kd*^. *k* is a constant, which scales the probability of cows from different locations to be in contact and has a unit *km*^*-1*^, and *d* is distance between the origin and destination locations. We assumed three different values of *k*, 0.001, 0.01, and 0.1, to reflect low, medium, and high movement probability, respectively. However, the network was valid for any value of *k*. We modeled potential transmissions of RVFV that resulted from movement, therefore, an infected transferred cow to a new location can infect others via local mosquitoes at the destination location.

**Fig 3:**
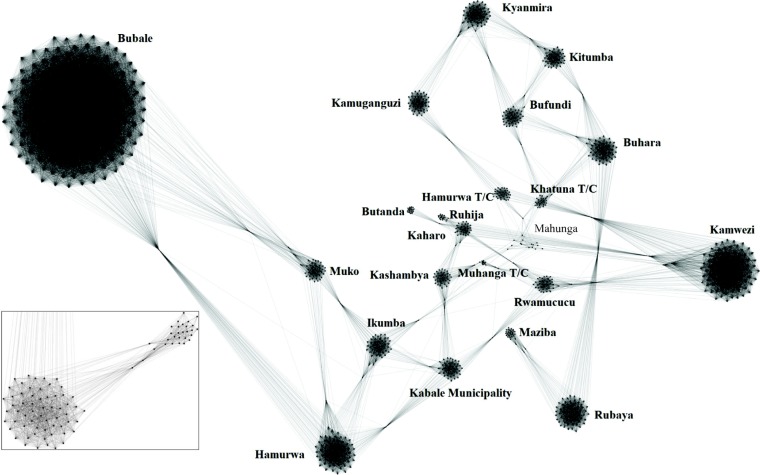
Overall structure of the network; dense circular groupings of black dots represent different locations. The inset shows a close-up of two such groupings, and one possible arrangement of links within and between them. Long black lines connect some locations, representing potential movement-related connections, and thus opportunities for mosquito-mediated transmission of RVFV between cows from different locations. The inset expands a small portion of the contact network showing the dense circular masses are made up of small black circles, each of which represents 20 cows and correspond to the nodes shown in the representative contact network. Likewise, the black lines among these nodes represent possible connections within and between locations in the inset.

We visualized 20,806 cows across the 22 locations using the network visualization software Gephi [31], but scaled cattle population sizes across the network by a factor of 1/20 for clarity and the example network is shown in **Fig 3**. It is important to note that scaling was only used for visualization and not model simulations, which were performed with the full value of *N*.

#### Cattle contact network scenarios

Cases in the literature indicated exotic cows showed more susceptibility to RVF than indigenous cows. Indigenous cows exhibited mild symptoms from RVFV infection and these cows might develop lower viremia, which could significantly affect transfer of the virus to mosquito vectors [32]. However, we do not have specific information on relative susceptibility of indigenous compared to exotic cows for the Kabale District in Uganda. This relative susceptibility could vary with breed as well as origin. Therefore, we assumed two different network scenarios to capture the relative susceptibility of exotic versus indigenous cow breeds while performing simulations with GEMF: a *homogenous* and a *heterogeneous* network.

In the *homogenous* network, we assumed that all cows, indigenous or exotic, have the same susceptibility to RVFV. Therefore, we used the total number of cows in each location rather than differentiating them in two different categories.

In the *heterogeneous* network, we assumed exotic cows were more susceptible to RVFV than indigenous cows. Lacking proper knowledge about the relative susceptibility, we assumed if exotic cows have a susceptibility *ζ*, then indigenous cows had a susceptibility of *μζ*, where *μ* had a value between zero and one. *μ =* 1 means a completely homogeneous network while *μ* = 0 means a network where indigenous cattle are immune to the RVFV pathogen. An increase in the value of *μ* from the minimum would increase network homogeneity and vice versa. For simulation purposes, we assumed *μ* = 0.7, which indicated 30 percent less susceptibility of the indigenous cows than exotic. However, we used this value to demonstrate effects of heterogeneity in RVFV transmission in a qualitative manner. We have invariably used a susceptibility *ζ =* 1 for exotic cattle in this work. Therefore, a *homogeneous* network can be considered as a network of only exotic cattle (susceptibility *ζ = 1*).

Simulations were performed for a variety of initial outbreak conditions, such as single location versus multiple location outbreaks with varying cattle populations, transmission rates, and cattle movement probabilities. We configured the model to investigate and quantitatively evaluate relative impacts of mosquito control, livestock movement regulations, and diversity in cattle populations. We explored different simulation sets, each consisting of a number of simulation scenarios. For each scenario, we performed 100 simulations.

We presented simulation results for different values of *k* as well as two ranges of transmission rate *β*. We investigated the number of cows in different compartments in the *SEIR* model by choosing a set of values of *β* (0.001, 0.005, 0.01, and 0.03), and starting with an infected cow in the Kabale municipality for each simulation. We chose a medium cattle movement probability constant *k* = 0.01 to reduce the number of simulations.

We also conducted simulations with different locations for the initial infected cattle, as well as single-location and simultaneous multiple-location RVFV epizootic outbreaks. We configured the network with values of *k* = 0.01 and performed simulations for *β* = 0.001, 0.005, 0.01, and 0.03 to reduce the number of simulation scenarios.

## Results and discussion

### Simulation set I

In this set, simulations were initiated with a single infected cow in the Kabale municipality and three values of *k* (0.001, 0.01, and 0.1), two ranges of *β* (0.0001–0.005 or 0.001–0.048), and two network topologies (*homogeneous* and *heterogeneous*), producing four scenarios:

**Scenario 1:** *Homogenous* network and *β* range 0.0001–0.005
**Scenario 2:** *Homogenous* network and *β* range 0.001–0.048
**Scenario 3:** *Heterogeneous* network and *β* range 0.0001–0.005
**Scenario 4:** *Heterogeneous* network and *β* range 0.001–0.048

#### Scenario 1

**Fig 4:**
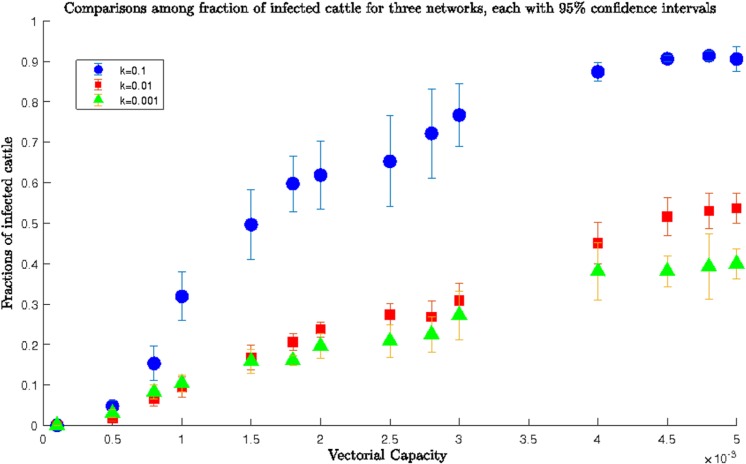
Comparisons among fractions of infected cattle for a *homogeneous* network for three different values of *k* and lower range of *β*; blue dots showed the fraction of infected for *k* = 0.1, while red rectangles and green triangles showed the fraction of infected for *k* = 0.01 and 0.001 respectively. For the same value of transmission rate, we always had more infected cattle for greater values of *k* (0.1) than the smaller ones (0.01 and 0.001). Therefore, increasing movement probability meant more widespread epizootic. For example, the fraction of infected cattle at *β* = 0,005 was ~0.399, ~0.537, and ~1 for *k* = 0.001, 0.01, and 0.1, respectively.

The simulation for *β* ranging between 0.0001–0.005 (the lower range) is presented in **Fig 4** for three different values of the exponential constant *k* and a *homogenous* network. We ran the simulation for 100 days and recorded the fraction of infected cattle for each value of *β*.

From **Fig 4**, for *k* = 0.01 and 0.001 it was evident that for *β =* 0.005, the infection reached half the population after 100 days. However, for *k* = 0.1, after 100 days almost all of the cows were infected as the network was densely connected and it was easier for RVFV to be transmitted between locations. A value of *k* = 0.1 meant extensive cattle movement between locations which made the whole network infected. Therefore, network structure played a prominent role in RVFV spreading when the value of *β* was small. Therefore, our simulation results conformed to the already established role of livestock movement and hence the network structure in the spread of RVFV [33].

#### Scenario 2

**Fig 5:**
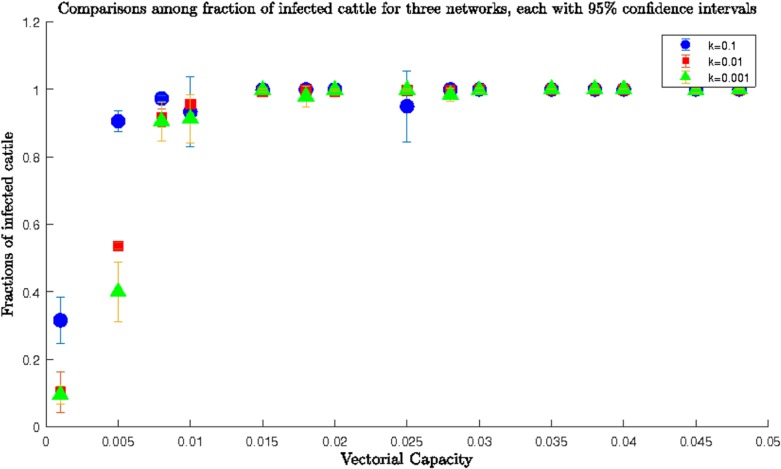
Comparisons among fractions of infected cows for *homogeneous* network for three different values of *k* and upper range of *β*; fractions of infected for all three values of *k* were almost overlapping, therefore, not sensitive to the movement probability. They reached a value very close to one, i.e., the whole network became infected when transmission rate *β* reached 0.01 for the three networks. Therefore, fractions of infected cows were also independent of the transmission rate.

In the second set of simulations, we used a *β* ranging from 0.001 to 0.048 for the *homogenous* network. Simulations results using these values are presented in **Fig 5** for all three values of the parameter *k*.

From **Fig 5** it was indicative the full network became infected very quickly for this particular range of *β* irrespective of movement probabilities (*k*). Because we already had connections between locations for all three networks, we could therefore say that for upper values of *β*, i.e., for higher abundance of mosquitoes and favorable weather conditions, the infection spread did not depend on the network structure and spread throughout the whole network very quickly [29].

#### Scenario 3

**Fig 6:**
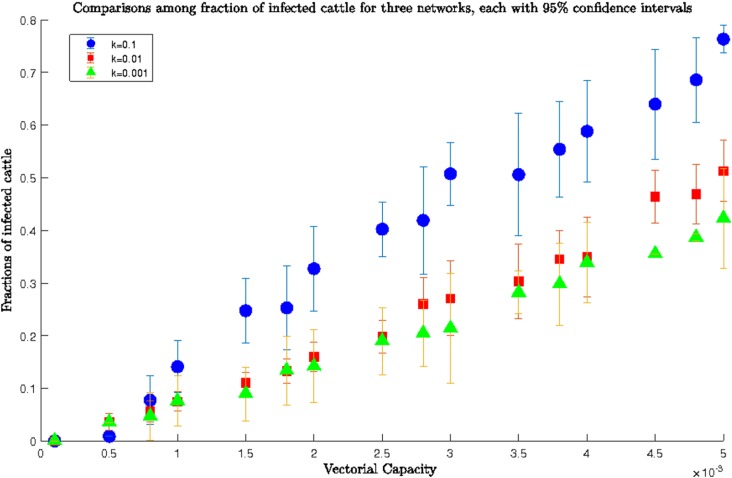
Comparisons among fractions of infected cows for *heterogeneous* network for three different values of *k* and lower range of *β*; for *k* = 0.001 and 0.01, the maximum fraction of infected cows was less than 0.5 for the highest value of transmission rate in the lower range, which meant after the simulation period half of the cows became infected. However, for *k* = 0.1, the infected cows reached up to 0.8. Therefore, we needed to reduce the value of *k* i.e., cattle movement, to reduce the fraction of infected cows.

In this scenario, we repeated simulations for the *heterogeneous* network and lower *β* range and presented simulation results in **Fig 6**. Trends increased in the fractions of infected cows with the increase of *β* as well as *k*. For *k* = 0.001 and 0.01, there was little difference; however, for *k* = 0.1 the increase of the infected fraction was faster with increasing *β*. Therefore, cattle movement needed to be reduced during an epidemic outbreak [34].

#### Scenario 4

**Fig 7:**
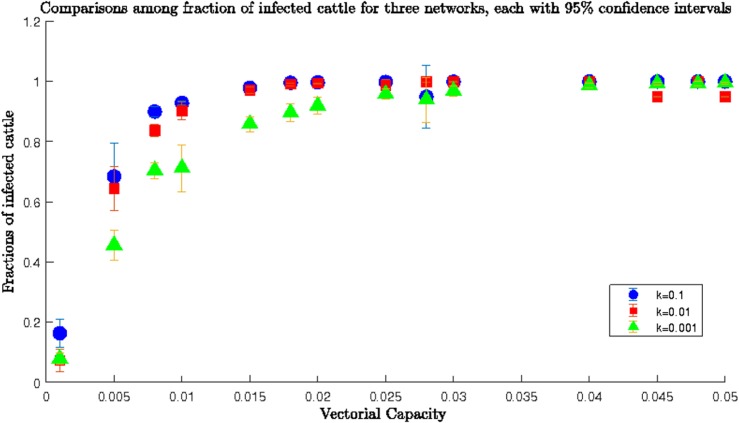
Comparisons among fractions of infected cows for *heterogeneous* network for three different values of *k* and for upper range of *β*; the fractions of infected reaches towards 1 rapidly and when the value of transmission rate is 0.03, the fraction of infected become one for all three networks.

Simulation results for the *heterogeneous* network and for the upper range of *β* are shown in **Fig 7**. For all three values of *k*, the fraction of infected cows reached 1 very quickly, near a *β* value of 0.03. After that, all cows became infected regardless of the values of *β* and *k*.

**Fig 8:**
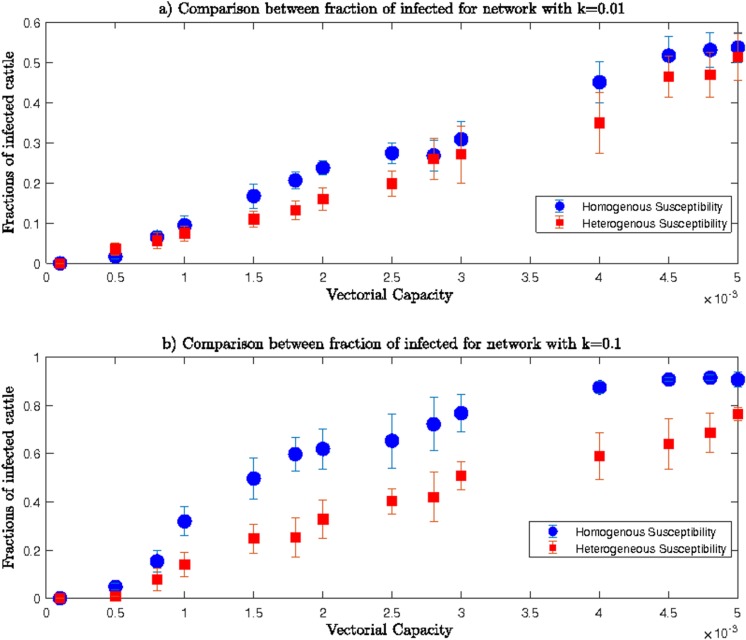
Comparisons among fractions of infected cows for heterogeneous and homogenous networks for lower range of β and a) k = 0.01 and b) k = 0.1.

**Fig 9:**
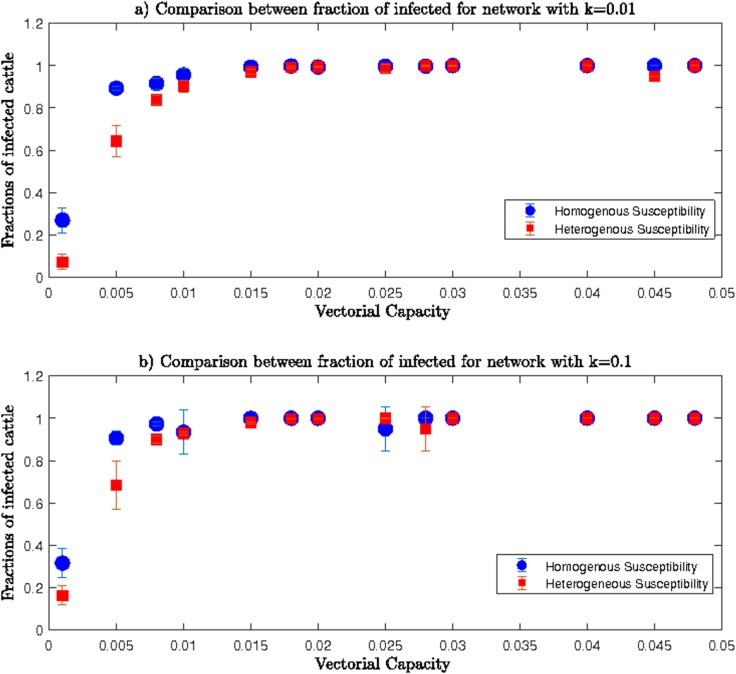
Comparisons among fractions of infected cows for *heterogeneous* and *homogenous* networks for the upper range of *β* and a) *k* = 0.01 and b) *k* = 0.1.

Trends of the fraction of infected cows for both *homogenous* and *heterogeneous* networks were similar in both lower and upper ranges of *β*. However, differences existed between fractions of infected cattle from *homogenous* compared to *heterogeneous* networks for the same value of *k* and the same range of transmission rate values. Comparisons between fractions of infected cows for *homogenous* and *heterogeneous* networks are shown in **Fig 8** and **Fig 9**.

**Fig 8** shows comparisons between fractions of infected cows for *homogeneous* and *heterogeneous* networks for lower range of *β* values, and shows the *homogenous* network had more infected cows for the same values of *β* compared to the *heterogeneous* network. Lesser susceptibility of indigenous cows resulted in fewer infections among them. Since we specified that indigenous cattle are less susceptible to infection, the *heterogeneous* network resulted in fewer infected cattle than the *homogenous* network where all cattle were exotic and highly susceptible.

In **Fig 9,** the difference between fractions of infected cows for two networks were negligible when *β*>0.00*5*. Therefore, lesser susceptibility of indigenous cows could not compensate for the higher mosquito abundance and results in similar infection spreading in both *homogeneous* and *heterogeneous* networks.

Comparisons between *homogenous* and *heterogeneous* networks showed reduced susceptibility of indigenous cattle meant a lower number of infected cows for lower mosquito abundance during an RVFV epizootic. Therefore, greater proportions of indigenous cows across locations would have the potential to reduce the numbers of infected cows and thus produce a more contained epizootic.

In summary, simulations with lower transmission rates resulted in increased fractions of infected cows with increasing movement probability. However, for high transmission rates, the fraction reached one and there was little difference in fractions of infected cattle while increasing movement probabilities. From these observations, we concluded that, for low transmission rates (low mosquito abundance), restricted cattle movement will reduce the number of infected cows. Higher transmission rates infected the whole network, regardless of cattle movement probability or mosquito abundance /transmission rate. Therefore, for a period of low mosquito abundance, cattle movement should be restricted to contain the epizootic to a minimum level; whereas, periods of high mosquito abundance (high transmission rates) would require both mosquito control and cattle movement restriction. Comparisons between fractions of infected for *homogenous* versus *heterogeneous* networks suggested that diversity in the network resulted in fewer infected cows for similar values of transmission rates and cattle movements.

### Simulation set II

Simulations were conducted starting with a single infected cow in the Kabale Municipality, using both *homogeneous* and *heterogeneous* networks with *k* = 0.01 and for *β =* 0.001, 0.005, 0.01, and 0.03 for each network, and produced two scenarios:

**Scenario 1:** *Homogenous* network
**Scenario 2:** *Heterogeneous* network

For each scenario, we assumed four different *β* to represent the entire range of transmission rates used in simulation set I. Instead of using different movement probability constants (*k* = 0.001, 0.01, and 0.1) we chose *k* = 0.01 for both *homogeneous* and *heterogeneous* networks.

#### Scenario 1

**Fig 10:**
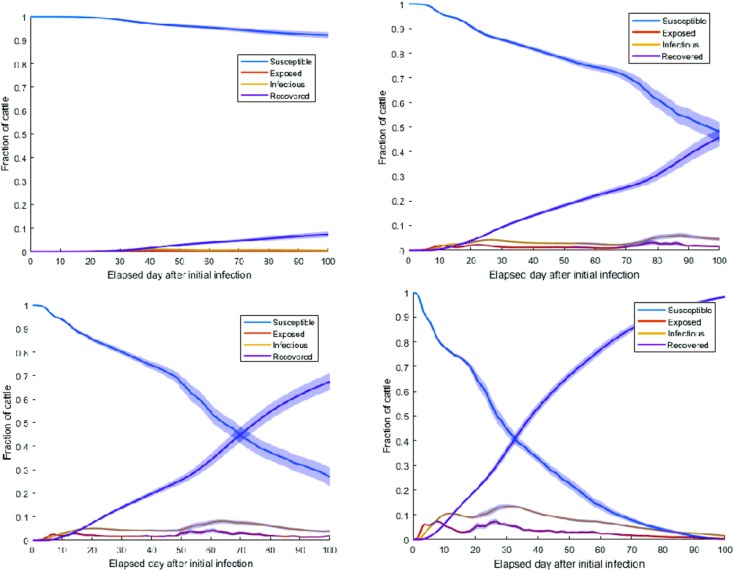
Fraction of cows in each compartment with a 95 percent confidence interval for *β* = 0.001 (top left), 0.005 (top right), 0.01 (bottom left), and 0.03 (bottom right) and for a *homogeneous* network; increasing *β* showed an increasing trend in the overall fractions of infected (cumulative fractions of recovered).

Simulation results for *homogenous* network and single infected cow in the Kabale municipality are presented in **Fig 10**. As *β* increased from 0.001 to 0.03, fractions of recovered reached 1 very quickly. It is worth noting that “fractions of recovered” means these were the cows that had been infected in the first place. As we had not considered any disease-induced mortality in the model, all infected cows moved to the recovered compartment. Therefore, the fraction of recovered cows was considered the cumulative fraction of infected cows for our specific model.

#### Scenario 2

**Fig 11:**
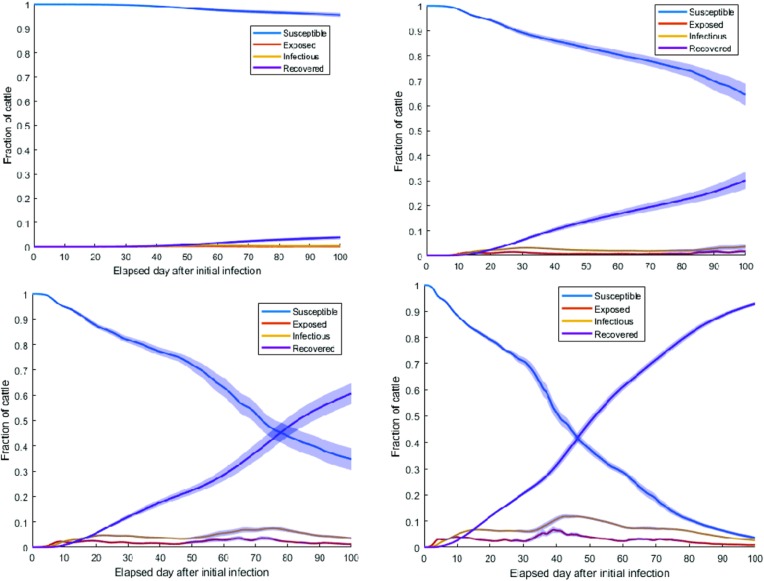
Fraction of cows in each compartment with a 95 percent confidence interval for *β* = 0.001 (top left), 0.005 (top right), 0.01 (bottom left), and 0.03 (bottom right) and for *heterogeneous* network; increasing *β* showed an increasing trend in the overall fractions of recovered (cumulative fractions of infected) which reaches to almost 1 for *β* = 0.03.

Simulation results for a *heterogeneous* network with the initial condition of a single infected cow in the Kabale municipality is presented in **Fig 11**.

From **Tables 2** and **3**, it was evident that with the increase of *β*, the rate at which the fraction of infected reached the maximum increased. However, a trend appeared that when the value of *β* was very small, i.e., *β*<0.005, the fraction of infected reacheed the maximum faster for both *homogeneous* and *heterogeneous* networks than higher values of *β (β>0*.*005)*. This can be attributed to the fact that, when the value of *β* was very small, the infection took a long time to reach distant locations. Therefore, cows in the Kabale Municipality became infected within our simulation period of 100 days and infection did not reach to distant locations, reinforcing the impact of reduced vectorial capacity in containing the outbreak. When *β* increased, infection reached distant locations at a slower rate than the rate of infecting animals only in the initial location. However, when infection reached distant locations, greater numbers of infected cows appeared in the network as a whole. This was evident from the maximum fraction of cows, which was greater than the maximum fraction of infected cows for *β* = 0.001. However, when *β* was large (0.03), the time to reach maximum was less than the time taken for *β* = 0.001. Simulation results indicated that an increase in the transmission rate expedited the spread of the epizootic in distant locations as well as the quantity of infected cows. Therefore, mosquito control was crucial to contain the epizootic in the initial outbreak location while taking proper measures to care for infected cows.

**Table 2:** Table shows maximum infected fractions of cows, peak infection time, and rate at which that maximum is attained for a *homogeneous* network.

Transmission rate *β*	Maximum infected fraction	Peak infection time	Rate
0.001	0.0095	45	2.1268e-04
0.005	0.065	87	6.919e-04
0.01	0.0806	64	0.0013
0.03	0.1345	31	0.0043

**Table 3:** Table shows maximum infected fractions of cows, peak infection time, and rate at which that maximum is attained for a *heterogeneous* network and a single infected cow in the Kabale municipality.

Transmission rate *β*	Maximum infected fraction	Peak infection time	Rate
0.001	0.0056	60	9.333e-05
0.005	0.0365	100	3.6479e-04
0.01	0.0739	76	9.7690e-04
0.03	0.1181	43	0.0027

### Simulation set III

This simulation set consisted of the following four scenarios:

**Scenario 1:** Infection began at a single location (Bubale) with maximum number of cows
**Scenario 2:** Infection began simultaneously at three locations (Bubale, Rubaya, and Hamurwa) with maximum number of cows
**Scenario 3:** Infection began at a single location (Muhanga T/C) with minimum number of cows
**Scenario 4:** Infection began simultaneously at three locations (Bukinda, Muhanga, and Ruhija) with minimum number of cows

**Fig 12:**
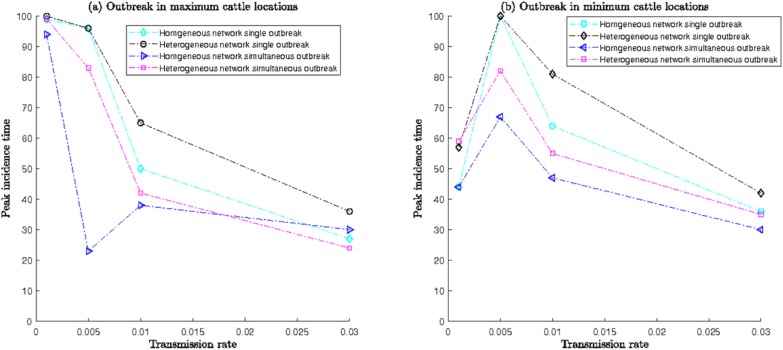
Peak infection time with transmission rate and for outbreaks starting in location/locations with (a) greater number of cows and (b) fewer number of cows.

For detailed descriptions of simulation results from Simulation set III, please refer to S1 Appendix. The time to reach maximum infection for each scenario with transmission rate *β* is shown in **Fig 12**, which shows a brief summary of *Scenario 1* and *2* simulations when the initial RVF outbreak occurred in a single location or simultaneously at multiple locations, respectively.

The time required to reach maximum infection was shorter for simultaneous outbreaks, regardless of the network structure, than for single-location outbreaks for similar values of the transmission rate *β* (**Fig 12A**). The spreading of infection through the network was slower in the *heterogeneous* network for both single and simultaneous outbreaks. Infections spread slowly for the single-location outbreak in the network compared to the rate of spread in simultaneous outbreaks, which was reflected by the higher peak incidence time. For *β* = 0.001, peak infection time was close to 100 days for all simulations except simultaneous outbreaks in *homogeneous* networks (**Fig 12A**). This means the peak was not attained, and the number of infected cattle was exponentially increasing. When *β* was increased to 0.01 (high mosquito abundance), the time to reach the peak reduced drastically for all of both single and simultaneous outbreaks.

When the value of transmission rate increased to 0.005–0.03, there was a correlated decrease in peak incidence time as the whole cattle network became infected very quickly (well before 100-day simulation period) irrespective of outbreak location(s). Outbreaks in locations with greater number of cows resulted in simultaneous virus introduction to distant locations having numerous connections. Therefore, with the increase in mosquito abundance, peak infection time decreased accordingly.

**Fig 12B** represents peak incidence time when the RVF outbreak occurred in location(s) with fewer cows than other locations. For lower mosquito abundance (*β* = 0.001), the infection did not reach distant locations, rather it was quickly confined to the initial location(s), as was evident from smaller values of the peak infection time. However, with increasing *β*, the peak time returned to its regular pattern shown in **Fig 12A**.

## Conclusions

When a RVF outbreak occurred in a location with many cows, the infection spreads faster while infecting greater numbers of cows than when an outbreak occurred in a location with fewer cows. Simultaneous outbreaks in multiple locations resulted in a more severe and faster-spreading epizootic than an outbreak in a single location. Given the same initial conditions, the *heterogeneous* network (different susceptibility of indigenous and exotic cows) was less susceptible to infection than the *homogeneous* (similar susceptibility of all cows) network. This was evident from the rate at which the infection reached maximum fraction of infected, the value of the maximum infected fractions, and total cumulative fraction of infected.

During periods with reduced vectorial capacity, prohibition of inter-location cattle movement eventually contained the epizootic to the outbreak location(s). There was an increased likelihood of more extensive RVFV transmission for upper transmission rates irrespective of the network structure. Control of mosquitoes became critical for elevated transmission rates to prevent widespread RVFV spread. The same level of transmission rates and inter-location movement probability resulted in fewer infected cows in *heterogeneous* networks than *homogenous* ones. Therefore, indigenous cattle provide protective herd immunity against RVFV hence minimizing outbreaks.

The rate of infection increased with an increase of the transmission rate, as well as the value of cattle movement probability. Simulation results from different initial starting locations showed that simultaneous outbreaks in different locations resulted in more infected cows at a faster rate of spreading compared to a single-location initial outbreak. The simulations showed how long the infection took to reach maximum for different network structures and conditions. A longer time to reach maximum infection spreading provided more time for applying mitigation strategies (mosquito control, culling/removing infected cows etc.) before the infection became widespread.

## Supporting information

S1 Appendix Supporting document- “individual-based network model for rift valley fever in Kabale District, Uganda”. This file contains all supporting figures and tables.
(DOCX)
