# Supplementary material for: Individual-based network model for Rift Valley fever in Kabale District, Uganda
Source: PLoS One. 2019 Mar 5;14(3):e0202721. doi: 10.1371/journal.pone.0202721 (PMC6400412; doi:10.1371/journal.pone.0202721)
Supplement: S1 Appendix — This file contains all supporting figures and tables. (DOCX) [file pone.0202721.s001.docx]

**S1 Appendix. *Supporting document- “*Individual-Based Network Model for Rift Valley Fever in Kabale District, Uganda*”.*** *This file contains all supporting figures and tables.*

**Graphical Representation of Inter-location cattle Movement**

Increasing the value of the scaling factor *k* in the equation e*^-kd^* , the probability of cattle movement among locations increases. This is demonstrated in Fig A, where we explored a wide range of value of *k*, and presented some of the network diagrams to demonstrate the change in the cattle movement with increasing value of *k.*


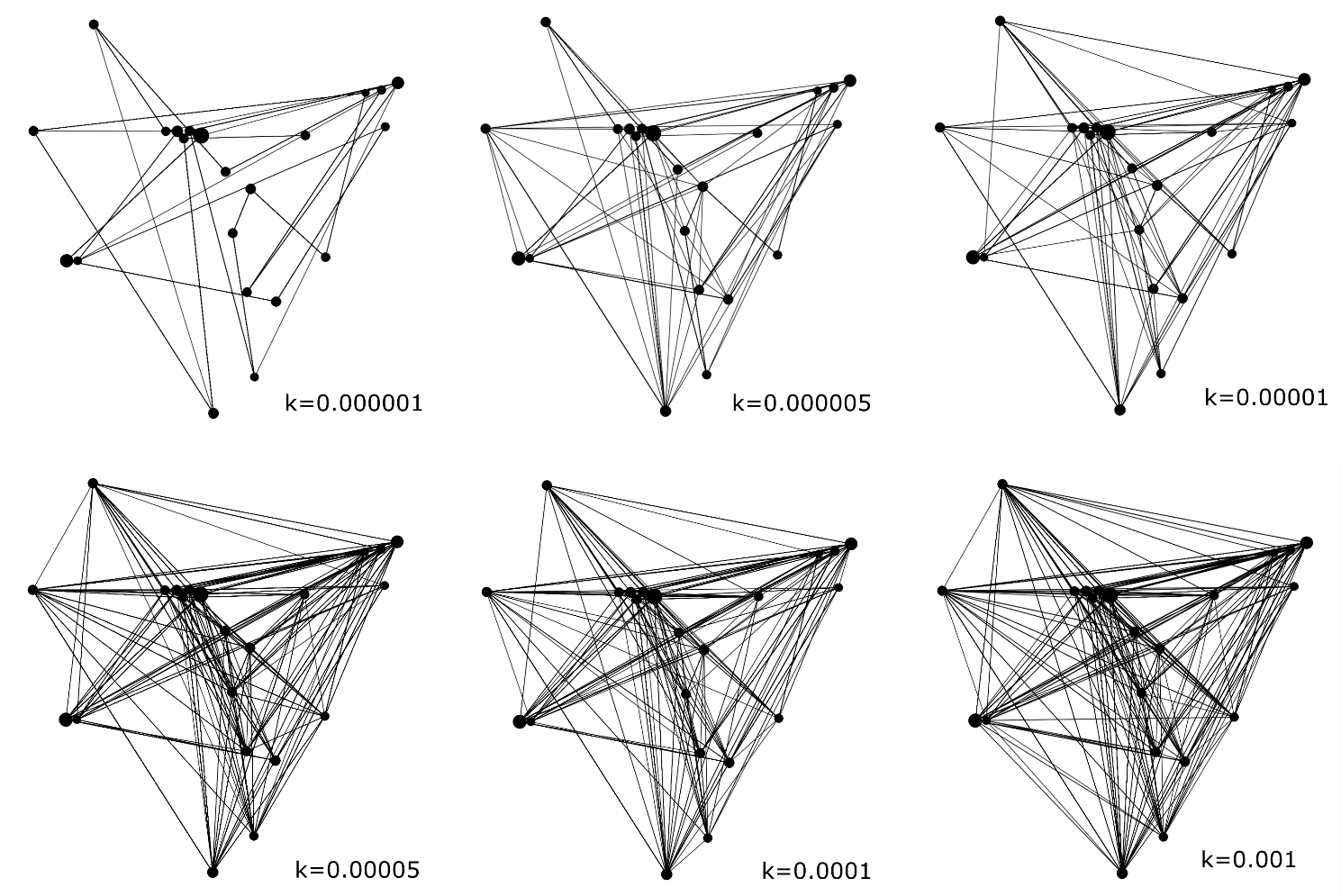


**Fig A.** **Inter-location cattle movement in Kabale district. Black dots represents each of 22 locations in the network, lines between dots represents the movement of cattle among them. Lines among dots (locations) keep increasing with *k*. Therefore, increasing the value of *k* increases the cattle movement.**

**Simulation SET III**

While conducting simulations for different starting conditions, we choose four starting conditions. Simulation results with for them are presented under four different scenarios in the subsequent part of this section.

**Scenario 1**

We start the simulation with a single infected cow at Bubale, which has the maximum number of cattle among our locations. The simulation results are shown in Figs B and C for *homogenous* and *heterogeneous* networks respectively. We present the rates of spreading infection, and time for reaching the maximum fraction infected in Tables D and E.

***Heterogeneous* Network**

Among our locations, Bubale sub-county has the maximum number of cattle (3301). A location that has maximum cattle has maximum links to other locations in the network. Therefore, to demonstrate the effect of the population size in the severity of an epizootic, we perform simulations with a single infected cow in Bubale sub-county for homogeneous network. Fig B represents fractions of cattle in different compartments for this scenario.
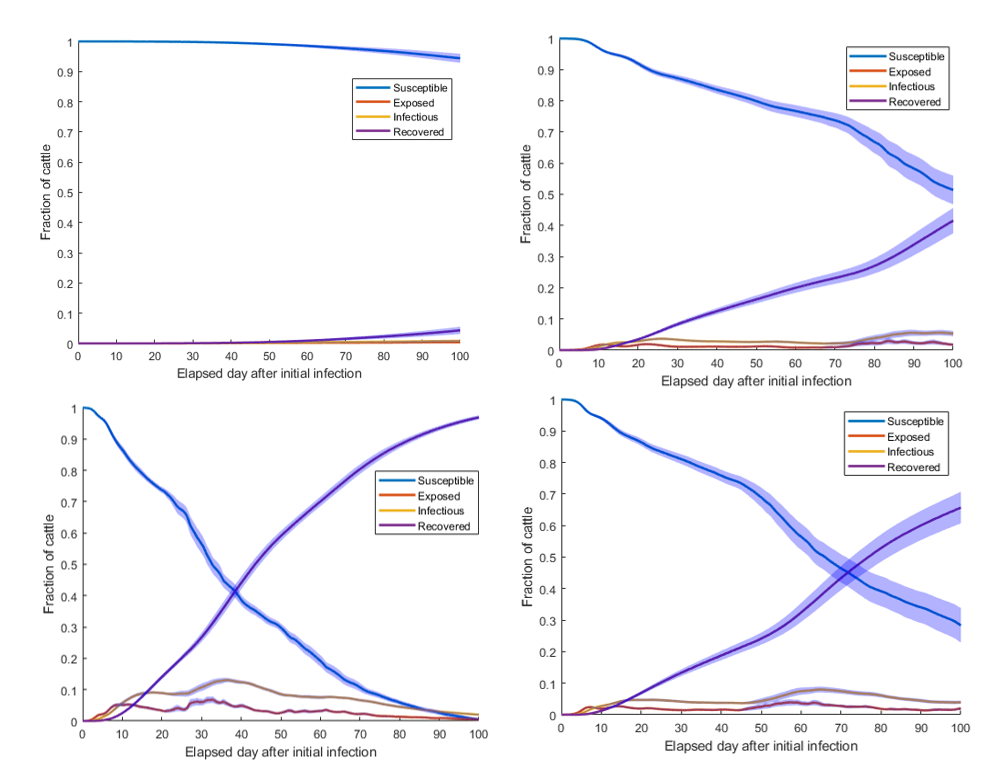


**Fig B. Fraction of cattle in each compartment with 95% confidence interval for *β*=0.001 (top left), 0.005 (top right), 0.01 (bottom left), and 0.03 (bottom right) and for *heterogeneous* network and infection starting at Bubale sub-county (3301 cattle).** Increasing *β* shows an increasing trend in the overall fractions of recovered (cumulative fractions of infected) which reaches to almost 1 for *β*=0.03.

**Table A. Table showing maximum infected fractions of cattle, peak infection time, and rate at which that maximum is attained for a *heterogeneous* network and single infected cow in Bubale sub-county.**

| **Transmission rate *β*** | **Maximum infected fraction** | **Peak infection time** | **Rate** |
| --- | --- | --- | --- |
| 0.001 | 0.0086 | 100 | 8.5864e-04 |
| 0.005 | 0.0562 | 96 | 5.8264e-04 |
| 0.01 | 0.0800 | 65 | 0.0012 |
| 0.03 | 0.1305 | 36 | 0.0036 |

***Homogeneous* Network**


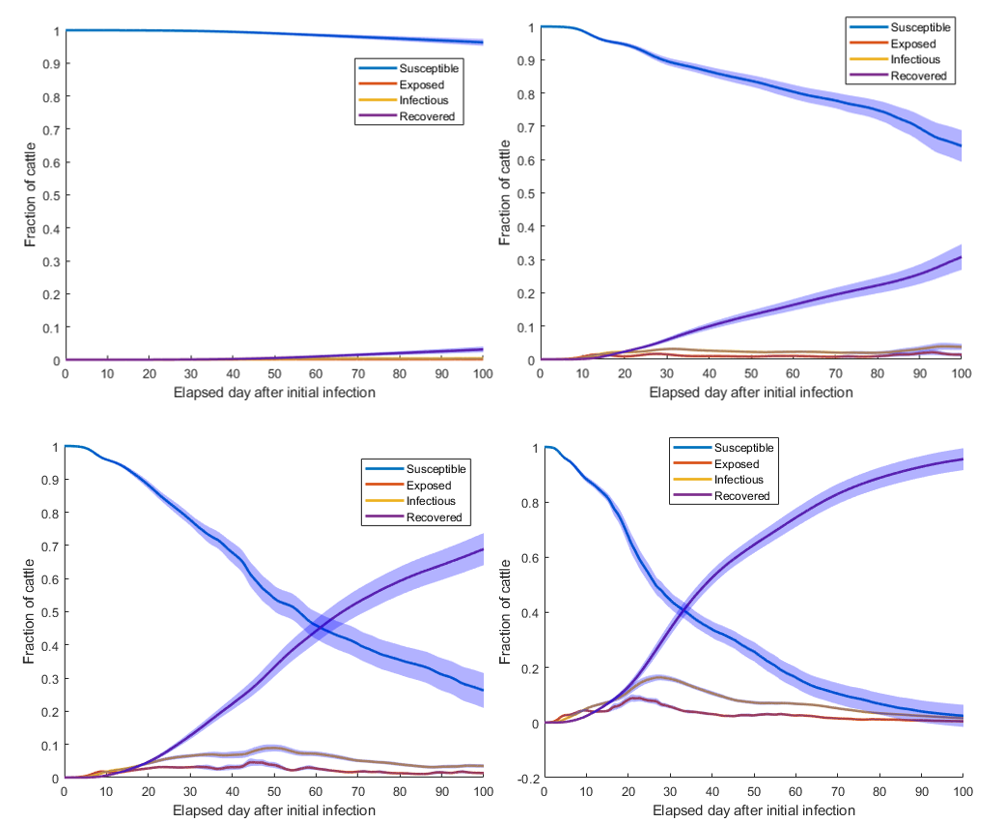


**Fig C. Fraction of cattle in each compartment with time with 95% confidence interval for *β*=0.001 (top left), 0.005 (top right), 0.01 (bottom left), and 0.03 (bottom right) and for *homogeneous* network and infection starting at Bubale sub-county (3301 cattle). Increasing *β* shows an increasing trend in the overall fractions of recovered (cumulative fractions of infected) which reaches to almost 1 for *β*=0.03.**

**Table B. Table showing maximum infected fractions of cattle, peak infection time and rate at which that maximum is attained for a *homogeneous* network and single infected cow in Bubale sub-county.**

| **Transmission rate *β*** | **Maximum infected fraction** | **Peak infection time** | **Rate** |
| --- | --- | --- | --- |
| 0.001 | 0.0041 | 99 | 4.1356e-05 |
| 0.005 | 0.389 | 96 | 4.0437e-04 |
| 0.01 | 0.0893 | 50 | 0.0018 |
| 0.03 | 0.01638 | 27 | 0.0059 |

From the comparison of B and C Tables, we can see that, the time for reaching the maximum fraction infected is always greater in the *heterogeneous* network than the *homogenous* one for similar values of the transmission rate. This time to reach the maximum fraction infected is crucial for public health personnel to plan in allocating resources to mitigate an epizootic spread. The time decreases with the increase of the transmission rate, therefore the mosquito abundance should be reduced to slow down the epizootic as well as gaining time for mitigation measures to come into effect. When infection starts at the location of maximum cattle, the infection keep spreading to the distant locations however slowly and that is evident from the time to reach the maximum fraction infected being close the ending time to the simulation period for *β* =0.001. Therefore, it is evident that times taken for reaching the maximum infection for other values of *β* are smaller than the time when infection starts at Kabale municipality for similar networks, therefore resulting in a widespread epizootic.

**Scenario 2**

Infection starts at the location with minimum number of cattle that is Muhanga T/C. Simulation results are shown in Figs D-E and Tables C-D for *homogeneous* and *heterogeneous* networks respectively.


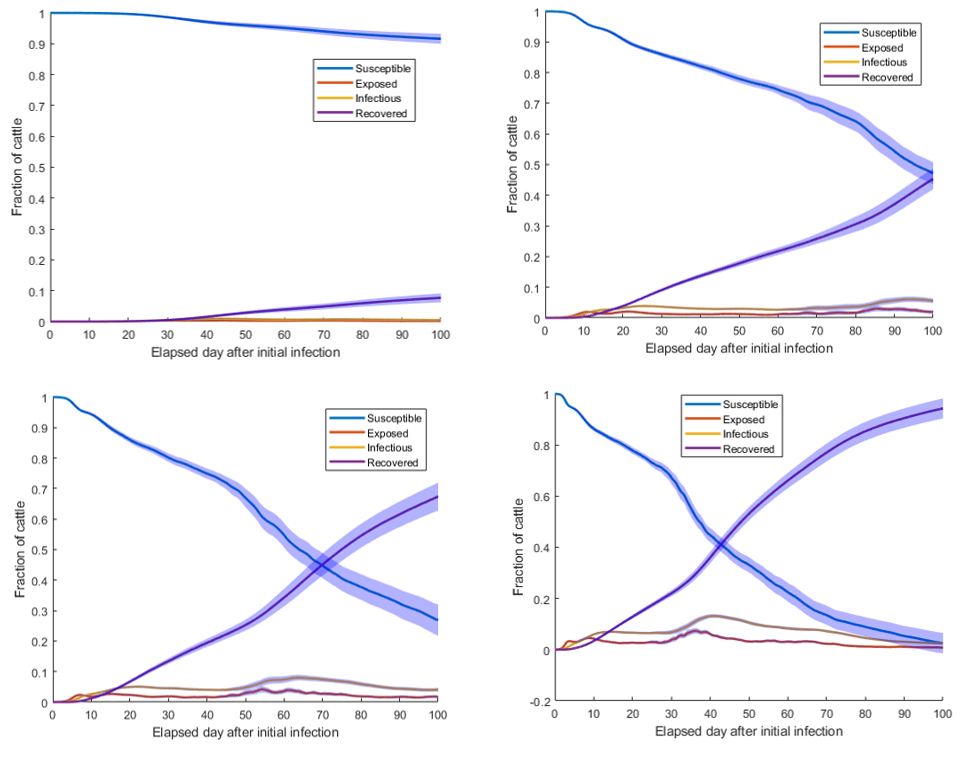


**Fig D. Fraction of cattle in each compartment with time with 95% confidence interval for *β*=0.001 (top left), 0.005 (top right), 0.01 (bottom left), and 0.03 (bottom right) and for *homogeneous* network and infection starting at Muhanga T/C (318 cattle).** Increasing *β* shows an increasing trend in the overall fractions of recovered (cumulative fractions of infected) which reaches to almost 1 for *β*=0.03.

**Table C. Table showing maximum infected fractions of cattle, peak infection time and rate at which that maximum is attained.**

| **Transmission rate *β*** | **Maximum infected fraction** | **Peak infection time** | **Rate** |
| --- | --- | --- | --- |
| 0.001 | 0.0097 | 44 | 2.2864e-04 |
| 0.005 | 0.0503 | 100 | 5.0484e-04 |
| 0.01 | 0.0802 | 64 | 0.0012 |
| 0.03 | 0.1305 | 36 | 0.0036 |

**Heterogeneous Network**


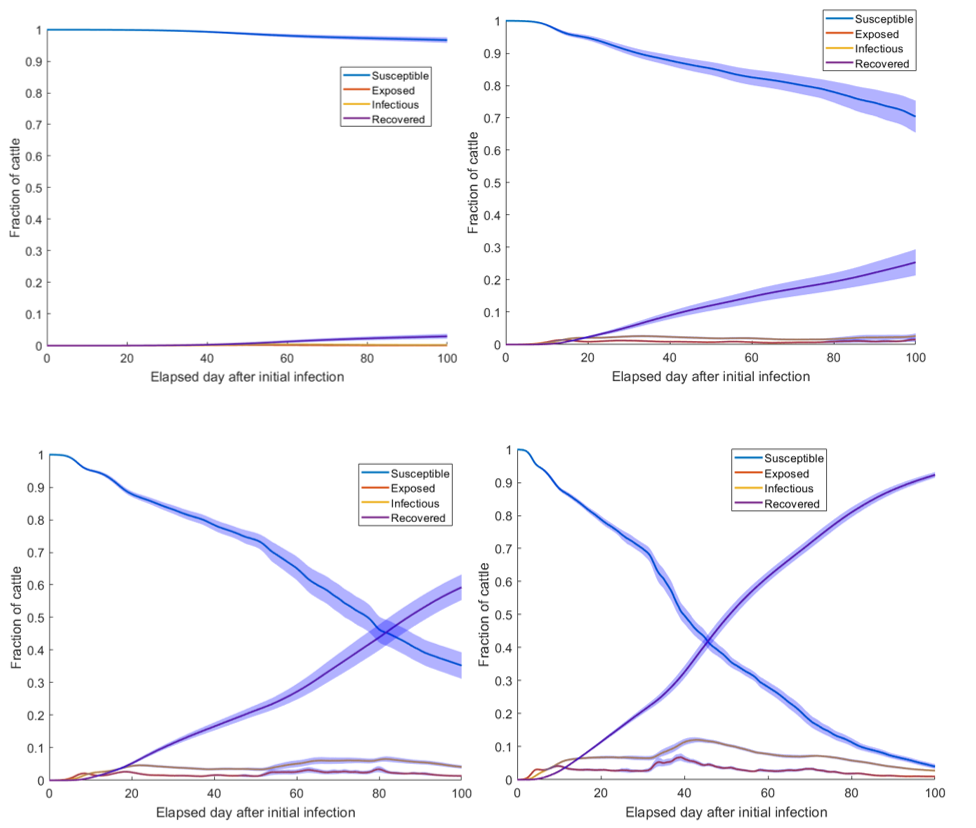


**Fig E. Fraction of cattle in each compartment with time with 95% confidence interval for *β*=0.001 (top left), 0.005 (top right), 0.01 (bottom left), and 0.03 (bottom right) and for *heterogeneous* network and infection starting at Muhanga T/C (318 cattle).** Increasing *β* shows an increasing trend in the overall fractions of recovered (cumulative fractions of infected) which reaches to almost 1 for *β*=0.03.

**Table D. Table showing maximum infected fractions of cattle, peak infection time and rate at which that maximum is attained.**

| **Transmission rate *β*** | **Maximum infected fraction** | **Peak infection time** | **Rate** |
| --- | --- | --- | --- |
| 0.001 | 0.0043 | 57 | 7.550e-05 |
| 0.005 | 0.0265 | 100 | 2.6443e-04 |
| 0.01 | 0.0655 | 81 | 8.02914e-4 |
| 0.03 | 0.1638 | 42 | 0.0023 |

For *β*=0.001, the time to reach the maximum infected fraction is smaller than the time required for *β*=0.005 and 0.01 because the infection does not reach to distant locations in this case. This can be attributed to the lower mosquito abundance as well as limited links to distant locations from the Muhanga T/C. We can see that when the infection starts at the location of the minimum cattle, the time taken to reach the maximum fraction of infected is always greater than *Scenario 1* that means infection spreads slower in this case of initial outbreak in Mahunga T/C.

**Scenario 3**

To demonstrate the effect of RVF outbreak in multiple location simultaneously, we choose Bubale, Rubaya and Hamurwa Sub-counties and started our simulation with one infected cattle in each of these locations. These locations were not chosen randomly, rather based on the maximum number of cattle among all locations. Simulation results are presented in Figs E- F and Tables F- G.

**Homogeneous Network**


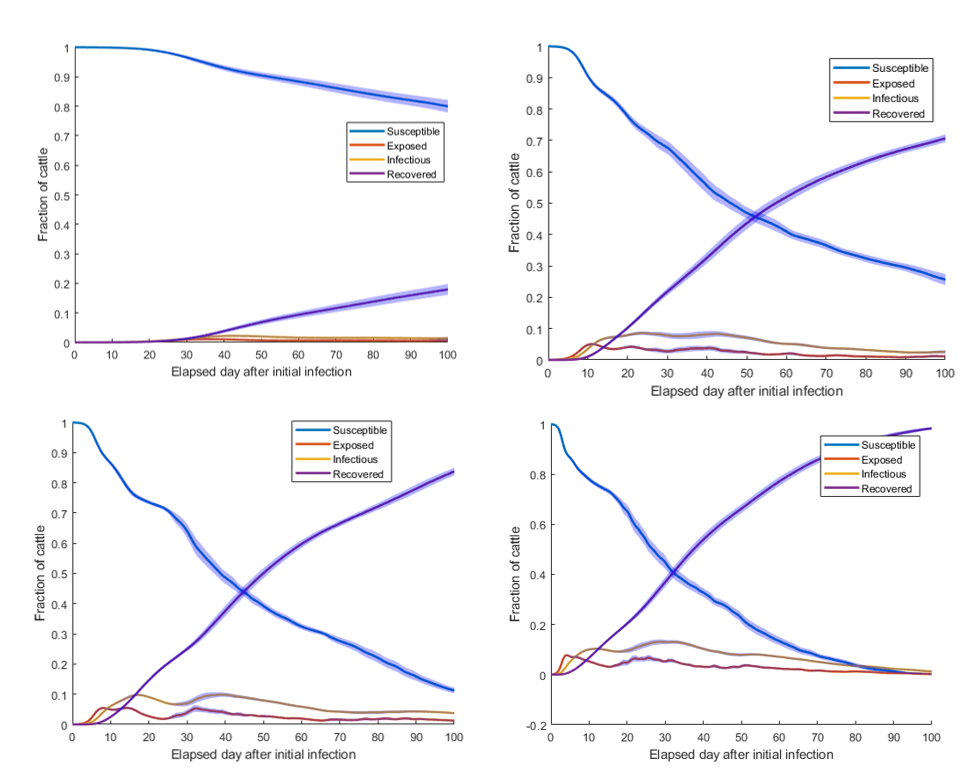


**Fig F. Fraction of cattle in each compartment with 95% confidence interval for *β*=0.001 (top left), 0.005 (top right), 0.01 (bottom left), and 0.03 (bottom right) and for *homogeneous* network and infection starting at Bauble, Rubaya and Hamurwa sub-counties.** Increasing *β* shows an increasing trend in the overall fractions of recovered (cumulative fractions of infected) which reaches to almost 1 for *β*=0.03.

**Table E. Table showing maximum infected fractions of cattle, peak infection time and rate at which that maximum is attained.**

| **Transmission rate *β*** | **Maximum infected fraction** | **Peak infection time** | **Rate** |
| --- | --- | --- | --- |
| 0.001 | 0.0129 | 94 | 1.36e-04 |
| 0.005 | 0.0845 | 23 | 6.45e-04 |
| 0.01 | 0.0986 | 38 | 0.0026 |
| 0.03 | 0.1307 | 29 | 0.0046 |


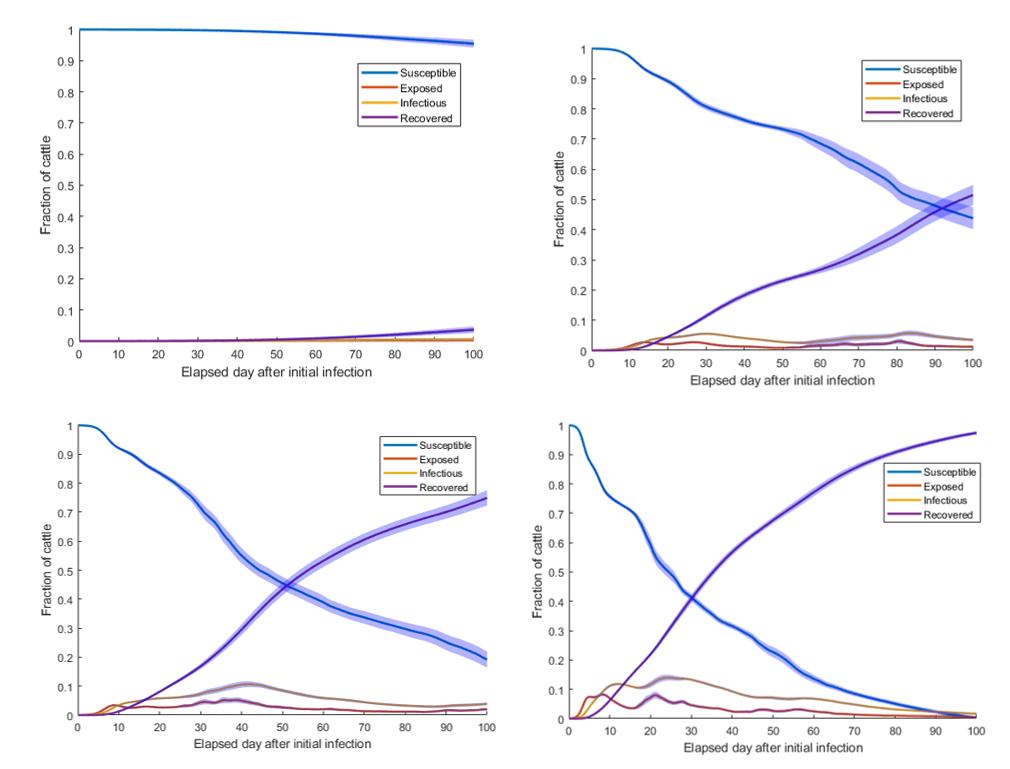


**Fig G. Fraction of cattle in each compartment with time with 95% confidence interval for *β*=0.001 (top left), 0.005 (top right), 0.01 (bottom left), and 0.03 (bottom right) and for *heterogeneous* network and infection starting at Bauble, Rubaya and Hamurwa Sub-counties.** Increasing *β* shows an increasing trend in the overall fractions of recovered (cumulative fractions of infected) which reaches to almost 1 for *β*=0.03.

**Table F. Table showing maximum infected fractions of cattle, peak infection time and rate at which that maximum is attained.**

| **Transmission rate *β*** | **Maximum infected fraction** | **Peak infection time** | **Rate** |
| --- | --- | --- | --- |
| 0.001 | 0.0061 | 99 | 6.1785e-05 |
| 0.005 | 0.0517 | 83 | 6.8687e-04 |
| 0.01 | 0.1065 | 42 | 0.0025 |
| 0.03 | 0.1398 | 24 | 0.0057 |

Tables E-F shows that, when we start the simulation with three location initially infected, we have faster increase in the fractions of infected than single initial infected location (*Scenario 1* and *2*). As we have seen before, the simulations results for heterogeneous networks always results in slower infection spreading rate than the homogeneous network for similar starting conditions and transmission rates.

**Scenario 4**

Simulations were conducted with Bukinda, Muhanga and Ruhija as the initial outbreak location. These locations were selected as they have fewer number of cattle than other locations. The simulation results for *homogeneous* and *heterogeneous* networks are presented in Figs H-I and Tables G- H.


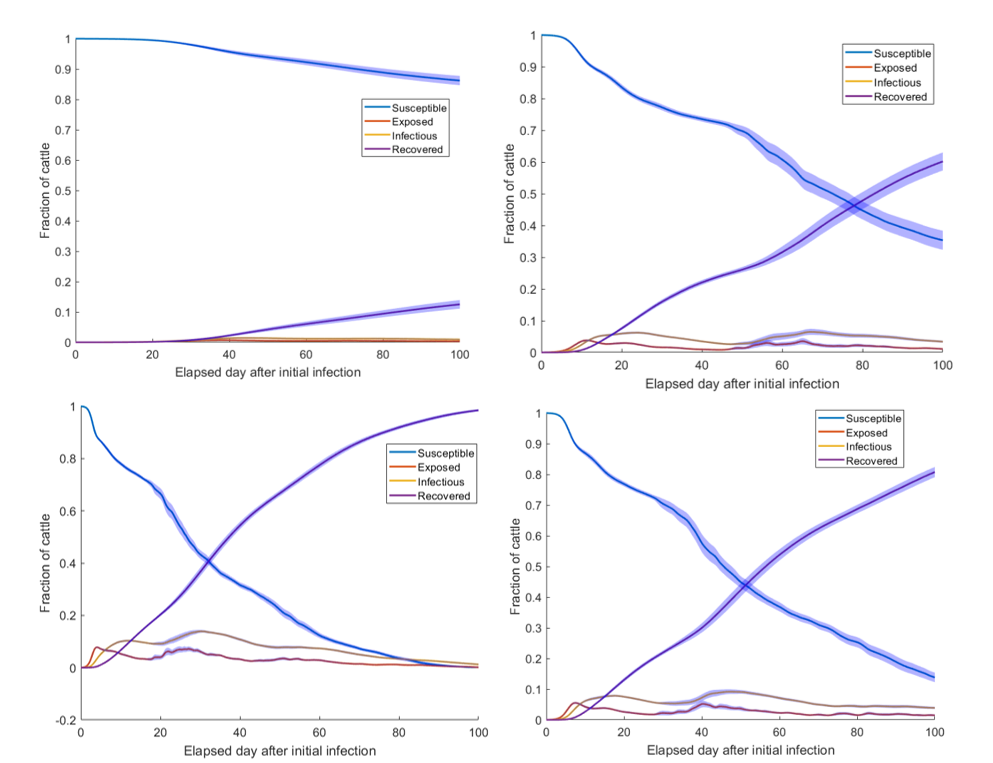


**Fig H. Fraction of cattle in each compartment with 95% confidence interval for *β*=0.001 (top left), 0.005 (top right), 0.01 (bottom left), and 0.03 (bottom right) and for *homogeneous* network and infection starting at Bukinda, Muhanga and Ruhija sub-counties.** Increasing *β* shows an increasing trend in the overall fractions of recovered (cumulative fractions of infected) which reaches to almost 1 for *β*=0.03.

**Table G. Table showing maximum infected fractions of cattle, peak infection time and rate at which that maximum is attained.**

| **Transmission rate *β*** | **Maximum infected fraction** | **Peak infection time** | **Rate** |
| --- | --- | --- | --- |
| 0.001 | 0.0145 | 44 | 3.2418e-04 |
| 0.005 | 0.0649 | 67 | 9.60e-04 |
| 0.01 | 0.0920 | 47 | 0.0019 |
| 0.03 | 0.1390 | 30 | 0.0046 |


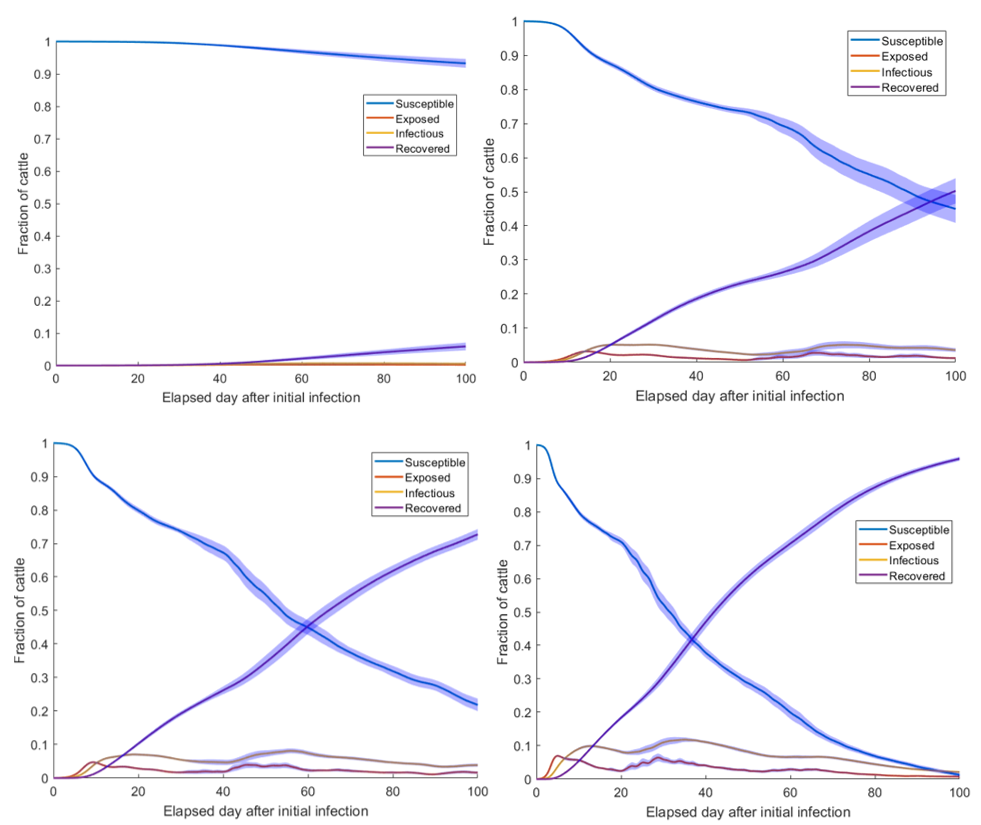


**Fig I. Fraction of cattle in each compartment with time with 95% confidence interval for β=0.001 (top left), 0.005 (top right), 0.01 (bottom left), and 0.03 (bottom right) and for *heterogeneous* network and infection starting at Bukinda, Muhanga and Ruhija sub-counties.** Increasing *β* shows an increasing trend in the overall fractions of recovered (cumulative fractions of infected) which reaches to almost 1 for *β*=0.03.

**Table H. Table showing maximum infected fractions of cattle, peak infection time and rate at which that maximum is attained.**

| **Transmission rate *β*** | **Maximum infected fraction** | **Peak infection time** | **Rate** |
| --- | --- | --- | --- |
| 0.001 | 0.0091 | 59 | 1.522e-04 |
| 0.005 | 0.0530 | 82 | 6.4e-04 |
| 0.01 | 0.0812 | 55 | 0.0015 |
| 0.03 | 0.1182 | 35 | 0.0033 |

As simulation started here in locations with fewer cattle than *Scenario 3,* infection spreading are slower than the case where infection starts at locations with greater number of cattle. Comparison among Tables E, F G, and H support this claim. When the value of *β*=0.001, infection becomes confined within the initial locations for our 100 day simulations period and results in a smaller time for the infection to reach maximum as infection does not reach to distant locations.

**Outbreak Scenarios**

**Scenario 1:** Homogenous Maximum cattle location single outbreak

**Scenario 2:** Homogenous Maximum cattle locations simultaneous outbreak

**Scenario 3:** Homogenous Minimum cattle location single outbreak

**Scenario 4:** Homogenous Minimum cattle locations simultaneous outbreak

**Scenario 5:** Heterogeneous Maximum cattle location single outbreak

**Scenario 6:** Heterogeneous Maximum cattle locations simultaneous outbreak

**Scenario 7:** Heterogeneous Minimum cattle location single outbreak

**Scenario 8:** Heterogeneous Minimum cattle locations simultaneous outbreak

**Table I. Table of Cumulative fractions of infected cows for all outbreak scenarios**

| **Scenario** | ***β*=0.001** | ***β*=0.005** | ***β*=0.01** | ***β*=0.03** |
| --- | --- | --- | --- | --- |
| **Scenario 1** | 0.05527 | 0.4436 | 0.6965 | 0.9755 |
| **Scenario 2** | 0.1976 | 0.7194 | 0.8576 | 0.9849 |
| **Scenario 3** | 0.09153 | 0.4799 | 0.7198 | 0.9821 |
| **Scenario 4** | 0.1392 | 0.6301 | 0.8241 | 0.9814 |
| **Scenario 5** | 0.04027 | 0.3433 | 0.7342 | 0.9752 |
| **Scenario 6** | 0.04745 | 0.5365 | 0.7233 | 0.9794 |
| **Scenario 7** | 0.0375 | 0.2927 | 0.6296 | 0.9147 |
| **Scenario 8** | 0.0711 | 0.5368 | 0.7438 | 0.9647 |

**Table J. Table of Cumulative number of infected cows for all outbreak scenarios**

| **Scenario** | ***β*=0.001** | ***β*=0.005** | ***β*=0.01** | ***β*=0.03** |
| --- | --- | --- | --- | --- |
| **Scenario 1** | 1150 | 9230 | 14492 | 20296 |
| **Scenario 2** | 4412 | 16466 | 17844 | 20492 |
| **Scenario 3** | 1904 | 9985 | 14977 | 20433 |
| **Scenario 4** | 2897 | 13111 | 17146 | 20419 |
| **Scenario 5** | 838 | 7143 | 15275 | 20290 |
| **Scenario 6** | 9782 | 11162 | 15048 | 20377 |
| **Scenario 7** | 781 | 6089 | 13099 | 19031 |
| **Scenario 8** | 1479 | 11168 | 15475 | 20071 |

**Network Metric**

**Node Degree**

For our work, we have not explicitly used the network metric. The gephi tool that we have used for network visualization cannot compute network metric for the actual network. We have developed a MATLAB code to calculate average node degree and the node degree distribution for the actual network.

An example of the is presented with the network while the value of *k*=0.001

The average node degree for the network =384.56

The node degree distribution is presented in Fig J.


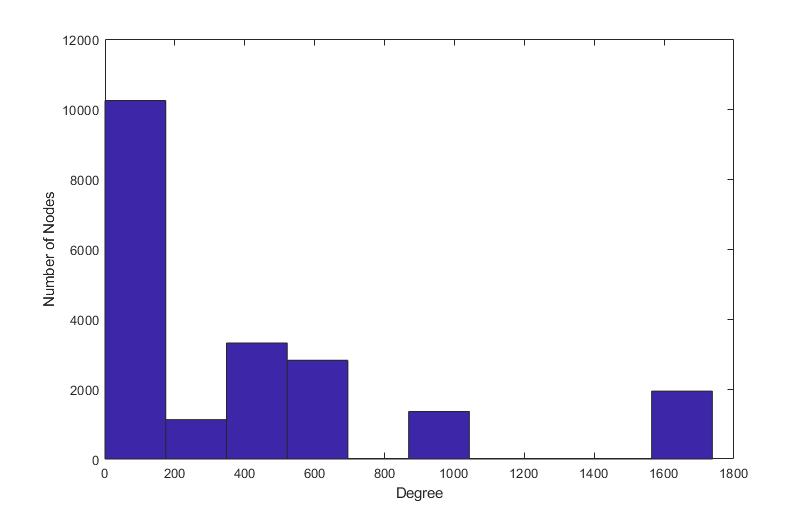


**Fig J. Node degree distribution for the network for the full network in Kabale district when k=0.001.**

**Cattle Contact Network**

The cattle contact network with the network topology is presented in Fig K.

**
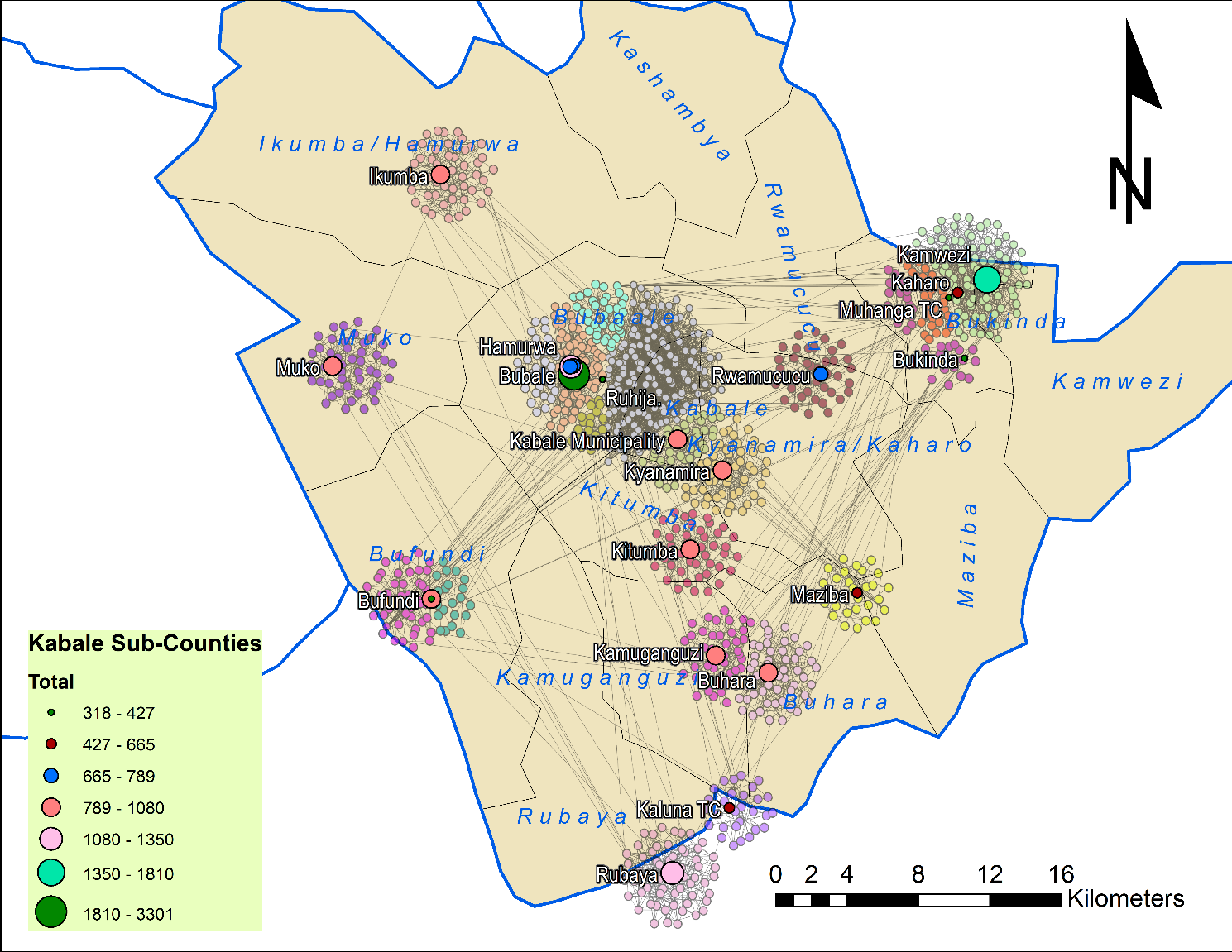
**

**Fig K. Locations of cattle contact networks in the Kabale District; circles represent center of each location and are color coded according to location and scaled according to total number of connections with other cows (degree). Point clusters in the underlying contact networks are distributed across a color ramp to visually differentiate cattle locations; these colors do not key into the scaled location centroids shown in the map legend.**
